# Characterizing Vancomycin-resistant Enterococci in Neonatal Intensive Care

**DOI:** 10.3201/eid1109.050148

**Published:** 2005-09

**Authors:** C. Rebecca Sherer, Bruce M. Sprague, Joseph M. Campos, Sumathi Nambiar, Rachel Temple, Billie Short, Nalini Singh

**Affiliations:** * Walter Reed Army Medical Center, Washington, DC, USA;; † Children's National Medical Center, Washington, DC, USA;; ‡ George Washington University School of Medicine, Washington, DC, USA;; § George Washington University School of Public Health, Washington, DC, USA

**Keywords:** Vancomycin-resistant enterococci, active surveillance, antimicrobial resistance, healthcare-associated infections, multidrug-resistant organisms, neonates, nosocomial infections, PCR, dispatch

## Abstract

Repetitive sequence–based polymerase chain reaction fingerprinting was used to characterize 23 vancomycin-nonsusceptible enterococcal isolates from 2003 to 2004. Five genetically related clusters spanned geographically distinct referring centers. DNA fingerprinting showed infant-to-infant transmission from referring institutions. Thus, community healthcare facilities are a source of vancomycin-nonsusceptible enterococci and should be targeted for increased infection control efforts.

Vancomycin-resistant enterococci (VRE) are a cause of nosocomial infections in US hospitals. The National Nosocomial Infections Surveillance system of the Centers for Disease Control and Prevention reported vancomycin resistance in 28.5% of nosocomial enterococcal intensive care unit infections in 2003 (*1*). In a recent study, 14% of VRE-colonized patients progressed to infection within 15 days of a positive surveillance culture (*2*). Moreover, VRE can transfer the *vanA* gene for vancomycin resistance to more virulent pathogens such as *Staphylococcus aureus* both in vitro and in vivo (*3*).

Risk factors for VRE colonization in children include young age, use of invasive devices, antimicrobial drug administration, immunosuppression, low birth weight, and underlying malignancy (*4*). Interfacility transfer of patients colonized with VRE is common, and previous hospitalization is a risk factor for harboring VRE at the time of hospital admission (*5**,**6*). Active surveillance culture (ASC) programs for VRE and aggressive implementation of infection control measures reduce VRE transmission among adult and pediatric patients (*7**,**8*).

We identified our first VRE-infected patient (bacteremia and urinary tract infection) in our neonatal intensive care unit (NICU) in 2000. This occasion prompted the initiation of our own ASC program. During the next 3 years (2000–2002), 65 patients with VRE colonization or infection were identified among the 1,820 patients admitted to our NICU. Of the VRE-colonized or -infected patients, some experienced serious infections, such as meningitis and bacteremia, while others were completely asymptomatic (*9*). By 2002, a multifaceted intervention greatly reduced the intrahospital spread of VRE. We continued our ASC program in the NICU during 2003–2004. An additional 25 patients were found to be colonized with vancomycin-nonsusceptible enterococci (VNSE); this group includes VRE and enterococci with intermediate susceptibility to vancomycin. All infants colonized with VRE had been admitted directly from regional hospitals in the Washington, DC, metropolitan area. This is the first study in which an ASC program and repetitive sequence–based polymerase chain reaction (Rep-PCR) fingerprinting were used to characterize the genetic relatedness and document intrahospital spread of VNSE.

## The Study

The NICU at Children's National Medical Center (CNMC) is a level III/IV 40-bed unit that provides care for critically ill patients in the first 6 months of life. CNMC has no obstetrics service; therefore, all NICU admissions are referrals from other hospitals. Our ASC program for VNSE included rectal swab cultures performed upon admission to the NICU. Repeat cultures were collected weekly from patients with negative admission cultures unless they became colonized or were discharged. Dacron-tipped swabs were moistened with sterile trypticase soy broth before rectal sampling. All neonates with VRE were placed on contact isolation during their NICU hospitalization.

Rectal swab specimens were added to *Campylobacter* blood agar plates containing 10 μg/mL vancomycin (Becton Dickinson Diagnostic Systems, Sparks, MD, USA). VRE were identified by using standard laboratory procedures. Species identification and vancomycin susceptibility were determined by using MicroScan Gram-Positive Breakpoint Combo Panels (Dade Behring, Deerfield, IL, USA) with a 24-hour incubation. Vancomycin susceptibility results were categorized according to the standards published by the Clinical and Laboratory Standards Institute (*10*). Susceptible isolates had vancomycin MICs <4 μg/mL, intermediate isolates had MICs 8–16 μg/mL, and resistant isolates had MICs >32 μg/mL. *Enterococcus faecium* and *E. gallinarum* were differentiated by using standard laboratory tests for motility and detection of acid production from xylose (*11*).

The genetic relatedness of 23 VNSE was determined with Rep-PCR DNA fingerprinting by procedures recently described (*12*). Briefly, DNA was extracted from 2 μL of an overnight VNSE culture by using the Ultraclean Microbial DNA Isolation Kit (Mo Bio Laboratories, Carlsbad, CA, USA). The extracted DNA was amplified by Rep-PCR by using the DiversiLab Enterococcus Kit (Bacterial Barcodes, Spectral Genomics, Houston, TX, USA).

The Rep-PCR products were analyzed by using the DiversiLab System and Software (Bacterial Barcodes, Spectral Genomics). The resulting DNA fingerprint patterns were viewed as virtual electropherograms. Analysis was performed with DiversiLab software version 2.1.6.6 by using Pearson correlation coefficients to determine genetic similarities and the unweighted pair group method with arithmetic mean to create dendrograms. Samples were classified into 3 groups: indistinguishable (similarity >97%), similar (similarity 95%–97% with fingerprint patterns displaying 1–2 band differences), and different (similarity <95% with >2 band differences) (*12*).

Of 1,333 NICU patients admitted during 2003–2004, a total of 25 were colonized with VNSE, yielding a colonization rate of 2%. The median age of these patients was 45 days (range 14–200 days). Twenty-three isolates were available for DNA analysis. Fifteen (65%) of the 23 isolates were *E. gallinarum*, and all had intermediate susceptibility to vancomycin. The remaining 8 isolates (35%) were *E. faecium* and all were vancomycin resistant. Rep-PCR analysis identified 5 distinct fingerprinting patterns with >95% similarities (Figure). The genetically related clusters are grouped as C1 (isolates 1–5, vancomycin-resistant *E. faecium*), C2 (isolates 9–12, vancomycin-intermediate *E. gallinarum*), C3 (isolates 13–14, vancomycin-intermediate *E. gallinarum*), C4 (isolates 15–20, vancomycin-intermediate *E. gallinarum*), and C5 (isolates 22–23, vancomycin-intermediate *E. gallinarum*). *Enterococcus* isolates 6, 7, and 8 (vancomycin-resistant *E. faecium*) and 21 (vancomycin-intermediate *E. gallinarum*) were genetically unique and unrelated to all other isolates tested. The similarity coefficients among members of each of the dominant clusters were >95% and the band differences were minor (i.e., only 1 or 2 band differences were found when gel images were compared with those of other cluster members). In cluster C1, 2 of the 4 isolates of *E. faecium* (isolates 1 and 2) had similarity coefficients >99% and were recovered from infants transferred from the same medical center. Both infants were admitted to our NICU within 2 weeks of each other. In cluster C4, 2 of 6 isolates of *E. gallinarum* (isolates 16 and 18) had similarity coefficients >98% and were recovered from patients transferred from the same medical center.

**Figure:**
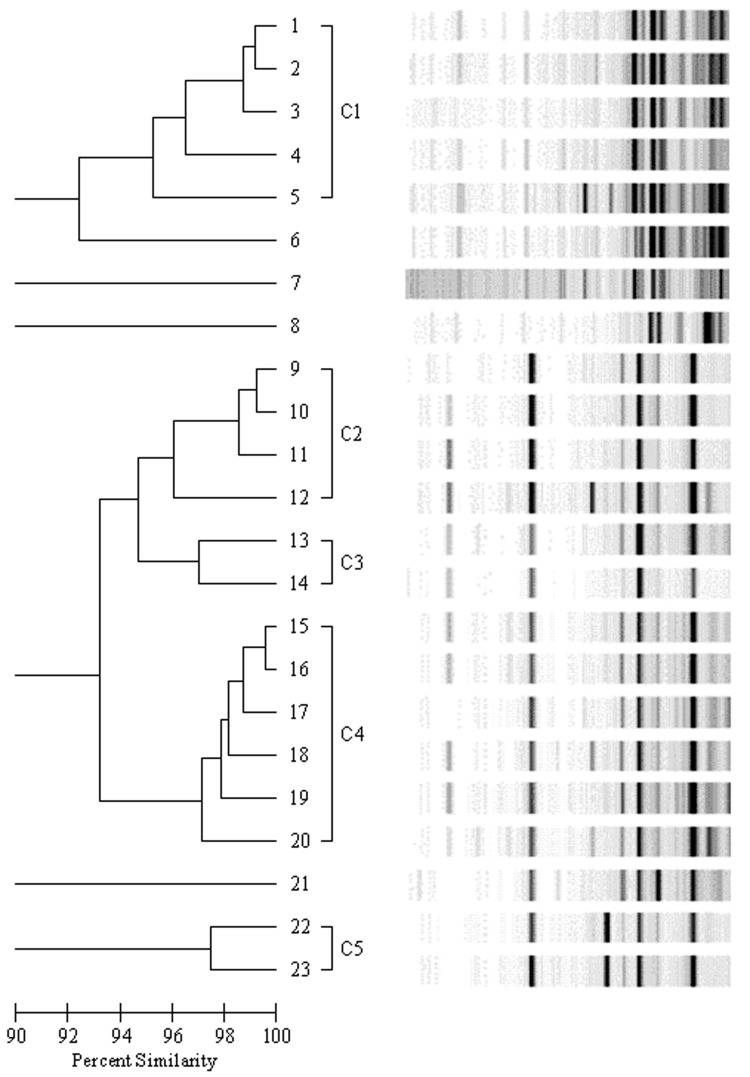
Dendrogram of 23 nonsusceptible enterococci isolates. Genetically related clusters are labeled C1–C5. Isolates represented by lanes 6, 7, 8, and 21 are genetically distinct.

## Conclusions

Because VRE can colonize the gastrointestinal tract for a prolonged period without progressing to clinically apparent disease, early recognition of colonization is essential for preventing patient-to-patient transmission. Of the 25 infants colonized with VNSE identified by our ASC program in 2003–2004, 23 isolates were available for DNA fingerprinting and further characterization. Thirty-five percent of the patients harbored vancomycin-resistant *E. faecium*, and 65% had vancomycin-intermediate *E. gallinarum*. Although *E. gallinarum* has low-level intrinsic vancomycin resistance and, thus, provokes fewer infection control concerns than high-level vancomycin-resistant *E. faecium*, invasive infections with this pathogen have been documented (*13*). The initiation of our ASC program, coupled with accurate and timely identification of VNSE, was associated with a sharp decrease in transmission of these bacteria to other NICU patients.

Molecular typing of VRE isolates with PCR and contour-clamped homogeneous electric field electrophoresis to conduct restriction fragment length polymorphism analysis of specific enterococcal genes has been described (*14*). These techniques enable targeted analysis of specific portions of the VRE genome and strain characterization. However, the overall genetic similarity of strains is unknown because only a small portion of the genome is assessed by these methods. Rep-PCR DNA fingerprinting is faster and easier to use than the other methods and results in a high level of genetic discrimination, making it a useful molecular epidemiologic tool.

Rep-PCR DNA fingerprinting identified 5 dominant clusters of VNSE in our NICU study patient population. Two instances of strong genetic relatedness were observed in isolates from neonates who were transferred from the same referral center within a limited period of time. The close relatedness of other VNSE was independent of the patient's hospital of origin. Infants from the same referral center usually did not have strains that were genetically related. One study described 7 VRE strains from 3 different locations within the same institution (*14*). Another study characterized VRE isolates from 6 different hospitals and found 23 isolates of 3 related types at 1 institution, while all isolates from another hospital were genetically distinct (*15*).

Referring centers that had transferred patients with VNSE to our NICU were informed of our results. As a result of our investigation, which showed no patient-to-patient transmission in our NICU during the study period, we established the following procedures: 1) we obtained ASC for VRE only from infants >14 days of age on admission to the NICU and placed them on contact isolation pending results, and 2) we no longer perform weekly surveillance cultures on previously culture-negative patients. This approach is cost-effective and sustainable.

Our ASC program identified a significant number of neonates admitted to our NICU who had been previously unrecognized as colonized with VNSE. Molecular fingerprinting of their isolates identified the existence of 5 clusters and several unique strains of VNSE circulating among newborns born in Washington, DC, metropolitan area hospitals. Our data also suggested that referring centers had experienced infant-to-infant spread based on similar Rep-PCR DNA fingerprint patterns. Our ASC program in tandem with the implementation of appropriate infection control measures led to a decrease in transmission of VRE to other NICU patients.
